# Treatment of Genetic Forms of Nephrotic Syndrome

**DOI:** 10.3389/fped.2018.00072

**Published:** 2018-03-26

**Authors:** Markus J. Kemper, Anja Lemke

**Affiliations:** ^1^AK Nord Heidberg, Asklepios Medical School GmbH, Hamburg, Germany; ^2^Universitätsklinikum Hamburg-Eppendorf, Hamburg, Germany

**Keywords:** steroid-resistiant nephrotic syndrome, mutations, cyclosporine, treatment, congenital nephrotic syndrome, Wilms tumor suppressor Gene 1, NPHS1, podocytes

## Abstract

Idiopathic steroid-resistant nephrotic syndrome (SRNS) is most frequently characterized by focal segmental glomerulosclerosis (FSGS) but also other histological lesions, such as diffuse mesangial sclerosis. In the past two decades, a multitude of genetic causes of SRNS have been discovered raising the question of effective treatment in this cohort. Although no controlled studies are available, this review will discuss treatment options including pharmacologic interventions aiming at the attenuation of proteinuria in genetic causes of SRNS, such as inhibitors of the renin–angiotensin–aldosterone system and indomethacin. Also, the potential impact of other interventions to improve podocyte stability will be addressed. In this respect, the treatment with cyclosporine A (CsA) is of interest, since a podocyte stabilizing effect has been demonstrated in various experimental models. Although clinical response to CsA in children with genetic forms of SRNS is inferior to sporadic SRNS, some recent studies show that partial and even complete response can be achieved even in individual patients inherited forms of nephrotic syndrome. Ideally, improved pharmacologic and molecular approaches to induce partial or even complete remission will be available in the future, thus slowing or even preventing the progression toward end-stage renal disease.

## Introduction

For many years, steroid-resistant nephrotic syndrome (SRNS), especially focal segmental glomerulosclerosis (FSGS) was thought to be an immunological disorder. This concept was supported by the response to immunosuppression in many patients and by the fact that recurrence after renal transplantation occurred, possibly due to the presence of a humoral factor, e.g., produced by the immune system (1) However, it is now known that a significant proportion of patients with SRNS [FSGS, but also diffuse mesangial sclerosis (DMS)] have an inherited cause of the nephrotic syndrome (NS), e.g., affecting structural proteins such as nephrin or podocin (2). At first sight, immunosuppression in such patients with inherited structural defects of the podocytes makes no sense. Yet, clinical observations, typically made in patients who were treated with immunosuppression as the genetic result was not yet available, show that a subset of patients does achieve partial or complete remission associated with such treatment. This raises the issue of optimal treatment in this cohort. Should all patients receive immunosuppressive treatment and if so, for how long and in what form? Are their clinical characteristics that can guide treatment? In general, prospective studies addressing this issue have never been performed and evidence pro -and contra- have been generated by retrospective series. Also, treatment options beyond immunosuppression (supportive and pharmacologic) have never been addressed prospectively, which is not surprising due to the rarity, severity, and heterogeneity of the diseases involved.

### General Considerations

Several factors have to be considered before choosing treatment for genetic causes of SRNS. First, the age of presentation and severity of initial symptoms is of utmost importance. In this respect, patients with congenital and infantile NS are probably the most problematic group, because children often present with severe symptoms, sometimes antenatally (3). Historically, the so called “Finnish” type of NS is a good example and aggressive treatment led to a dramatic improvement of survival and long-term-outcome (4). Although patients presenting with infantile NS (onset in the first year of life) have a high risk of carrying a monogenic mutation (5), it should be noted that some patients can have a good prognosis reaching remission with supportive treatment alone (6); individual patients may in fact have minimal change disease responding to steroids. Thus, not only genetic testing but also renal biopsy should be considered in this group.

Second, genetic testing results need to be considered for treatment. Although only mutations in few genes are frequent, there are now panels available testing for 30 or more genes and it can be assumed that there will be new genetic causes in the future. This implies a clinical heterogeneity, not only among identical but also between different genotypes. In this respect, it needs to be at least mentioned that there is often a delay in getting results of genetic testing, which often takes weeks, sometimes months (and sometimes years because new variants are to be detected). Some patients with a negative initial test result may have a yet undiscovered monogenetic cause, and this has implications in choosing a therapeutic approach.

The last problem arises from the fact that there is no universal consensus regarding the definition of treatment response. A (rapid) complete remission of proteinuria is the ideal situation and will generally be accepted without any discussion. However, the definition of partial remission is much more problematic. Fluctuations of proteinuria are difficult to evaluate as they can occur with and without treatment. Most authors would agree that a reduction of proteinuria, for instance, by 50% can be regarded as partial remission, others would demand a concomitant increase of serum-albumin with cessation of edema, which obviously is of greater clinical relevance. There is also no consensus on how to exactly assess the impact of treatment on glomerular filtration rate (GFR) and renal survival.

### Supportive and Non-Immunologic Treatment for Genetic Causes of NS

#### Congenital and Infantile NS

The prognosis of congenital NS has improved substantially in recent years. No differences in mortality and transplant outcome between Finnish and non-Finnish patients with *NPHS1* mutations was noted in a recent registry report on 170 patients (4). Finnish patients started dialysis much earlier because of early bilateral nephrectomy, while in non-Finnish, many other interventions were performed (but not reported on in detail). Despite this, outcome of *NPHS1* patients on renal replacement therapy in fact compared to patients with congenital anomalies of the kidney and urinary tract (CAKUT). No details of treatment approaches and mortality prior end-stage renal disease are presented however. These may influence morbidity and mortality in patients with *NPHS1* and other genetic causes, however and include the following options.

##### Albumin Infusions

In severe forms of congenital but also infantile NS regular (mostly daily) albumin infusions have been recommended to decrease edema, increase urine output, and enhance nutrition (7). This strategy requires sufficient renal function; otherwise, fluid overload may occur with potentially severe consequences, such as cardiac failure or pulmonary edema. Since infusions have to be performed regularly, often daily, a central venous access is usually necessary with the associated risks of infection, thrombosis, and hospitalization. Of interest, a recent report by Reynolds et al. (7) showed that after adequate training, administration of albumin can be performed at home, which has an important impact on quality of life. Unfortunately, in reports on regular albumin infusions, other treatments have been used as well (see below). In a yet unpublished French study, 96% of patients received albumin infusions initially daily, with a subsequent reduction in frequency in many patients. It was even discontinued in 10 patients. However, in this report, data about concomitant drug treatment are not available (Berody et al. accepted by NDT, complete citation expected to be available in February).

##### Nephrectomy

Unilateral or even bilateral nephrectomy has been used as therapeutic option to decrease or stop proteinuria. Bilateral nephrectomy is probably the most aggressive approach, which will on the one hand completely stop proteinuria, normalize protein and lipid status, and improve nutritional state, but on the other, make (peritoneal) dialysis treatment inevitable (3). Unilateral nephrectomy has been advocated by some authors to reduce proteinuria in children with congenital NS, again often in addition with medical treatment (indomethacin and captopril). In one study (8), serum albumin (sAlb) increased from 11 to 18 g/l after 6–12 months and the number of albumin infusions could be reduced later. This series of five patients also documented an increase in height standard deviation score.

##### Renin–Angiotensin–Aldosterone System (RAAS) Inhibitors (With/Without Indomethacin)

A further more conservative approach is drug treatment in order to reduce GFR and thus decrease proteinuria. The value of inhibiting the RAAS by ACE inhibitors (ACEI) and angiotensin receptor blockers (ARBS) in proteinuric renal diseases has been established for many years (9), starting from studies of IgA nephritis. Therefore, in clinical practice, RAAS inhibitors are widely used frequently even in the absence of hypertension. The mechanisms of action relate to decreasing intraglomerular pressure as well as anti-TGFβ properties leading to deceleration of the progression of renal insufficiency. The Cochrane group (9) included RAAS inhibitors in their recommendations for treatment of SRNS but no large studies concerning their use in congenital or infantile NS are available. In one (8), RAAS inhibitors were combined with indomethacin and unilateral nephrectomy. Licht et al. (10) used a stepwise approach: five patients with different causes of congenital NS were treated with captopril and indomethacin serum protein and growth improved in four children. Unilateral nephrectomy was only deemed necessary in two patients during the subsequent course.

Although published evidence is limited, these studies support a stepwise approach in a clinically stable patient with congenital/infantile NS starting with the use of RAAS inhibitors. In neonates and infants, captopril has been most frequently used and can be titrated best. In severe cases, a combination with indomethacin seems justified; if edema are controlled by this approach (unilateral), nephrectomy can be avoided.

#### Non-Immunologic Treatment in Pediatric and Adolescent Genetic SRNS

Presentation of SRNS after the neonatal period usually leads to a different approach, because the underlying genotype and histological lesions are typically different; *NPHS2* mutations are probably the most frequent single genetic cause (2). Bilateral or unilateral nephrectomy is only practiced in individual severe cases. Treatment with steroids usually has been initiated prior diagnosis and—per definition—failed. Since results of genetic testing are not available immediately in many patients, further treatment, e.g., with calcineurin-inhibitors will be considered and initiated, except maybe in syndromic SRNS. In most published registries (11–13), patients with genetic nephrotic syndrome had received immunosuppressive treatment.

As in congenital and infantile NS, treatment with RAAS inhibitors are a possibility to decrease proteinuria, slow progression of chronic kidney disease, and treat hypertension. RAAS inhibitors are often used in combination with immunosuppressants in glomerulonephrits and NS, although few systematic studies are available evaluating the isolated or combined treatment. As mentioned before, RAAS inhibitors are recommended by the Cochrane group (9) because of two studies. Yi et al. (14) treated SRNS patients with fosinopril and prednisone and compared to a group receiving prednisone alone. Proteinuria decreased significantly in both groups but more in the fosinopril-treated patients. Bagga et al. (15) studied the effect of enalapril at high and low doses showing that there is a dose-related reduction in proteinuria. The benefits of a combined treatment of ACEI and ARBS was suggested by small prospective study in eight patients with SRNS (16). One case report documented complete remission with captopril; regular albumin infusions could be stopped at the age of 15 months (17). Combined antiproteinuric therapy with RAAS inhibitors was also able to induce complete remission in a patient with Nail–Patella syndrome and NS (18). Although all these studies are small and data on genetic causes are not always provided, the long experience with RAAS inhibitors in children would probably be in favor for early use of these drugs in pediatric patients, especially since they are tolerated well and can also be used for associated hypertension. Nevertheless, more controlled data are desirable. Of interest, a current controlled study comparing sparsetan (a dual acting angiotensin receptor blocker and highly selective endotheline Type A receptor antagonist) with irbesartan has been initiated in FSGS to assess the impact also in genetic forms of NS (19).

## Other Options

In individual patients with FSGS (mainly with permeability factor associated NS), treatment with galactose had been reported to be beneficial. Trachtman et al. (20) evaluated treatment of FSGS with a TNF- α inhibiting antibody adalimumab and galactose, the latter being an intersting non-immunologic treatment option also for genetic forms of FSGS. In the cited study, 2 out of 7 patients with FSGS had a 50% reduction in proteinuria after galactose, confirming previous case reports. Data on genetic testing in patients are not available, so no definite conclusion about the utility of galactose in genetic SRNS can be drawn. Unfortunately, a controlled trial with a monoclonal anti-TGF-β antibody (fresolimumab) in SRNS did not lead to a significant reduction in proteinuria (21).

Similarly, the use of vitamin D analogs and stimulation of the calcium-sensing receptor has been assessed. Experimental studies have shown that stimulation of the calcium-sensing-receptor enhances podocyte stability and thus cinacalcet (or vitamin D) may be an option to improve proteinuria in NS (22). So far, data on cinacalcet are not available, but a recent meta-analysis in IgA nephropathy suggested an effect of vitamin D supplementation (23). However, a recent randomized controlled trial of vitamin D supplementation in steroid-sensitive NS did not reduce relapse rate, arguing against a direct podocyte stabilizing effect of vitamin D (24). Although definitive conclusions of these alternative approaches cannot be drawn, future studies into the field of podocyte stabilization by non-immunosuppressive drugs are warranted and could have an important impact on treatment in genetic SRNS.

### Immunosuppression in Hereditary SRNS

#### Evidence from Experimental Studies

Several experimental studies have suggested that immunosuppressants have a direct glomerular effect leading to podocyte stabilization and thus have efficacy beyond their immunological actions. Some agents, such as cyclosporine also have a hemodynamic (nephrotoxic) effect leading, e.g., to reduction of GFR, thereby reducing proteinuria (25).

The initial studies confirming a stabilization of the glomerular cytoskeleton by cyclosporine independent of the immunosuppressive action was provided by Faul et al. (26) and confirmed by various other studies that will not be described in detail (27). Also, steroids, levamisole, mechanistic target of Rapamycin (mTOR) inhibitors, and even Rituximab have received attention in experimental studies although clinical data are virtually non-existent. For instance, glucocorticoids have been shown to protect and enhance recovery of cultured murine podocytes *via* actin filament stabilization (28, 29). Levamisole, a drug that has never been used in SRNS, was able to induce expression of glucocorticoid receptor (GR) and to activate GR signaling and also protected against podocyte injury in a cell model (30). Also, low-dose rapamycin, an inhibitor of the mTOR, diminished disease progression in an experimental model of FSGS (31). Finally, rituximab, a B-cell-depleting antibody, may bind directly on sphingomyelin phosphodiesterase acid-like 3b protein (SMPDL3b), and thus could have an effect at the cellular level (32).

In summary, there is now emerging evidence that immunosuppressants, especially cyclosporine A (CsA), have a stabilizing effect at the podocyte levels aside from their immunological actions in experimental models and thus may be valuable therapeutic options in the treatment also of genetic forms of SRNS.

## Clinical Data on Immunosuppressants in Genetic NS

Most authors would agree that children with *NPHS2* mutations do not respond to treatment with steroids (33). However, in their first report on mutations in the *Plectin1* (*PLEC1) gene*, the authors mention two patients who responded to prednisolone treatment (34). This could not be confirmed from a French series (35); interestingly, the authors report on three unaffected and unrelated patients with homozygous PLCE1 mutations but without clinical disease. Thus, the natural history of SRNS with PLEC1 mutations may be different and modifier genes and environmental factors may play a role. There are no data, whether patients with SRNS and other mutations have a documented partial or complete remission after initial steroid treatment.

To our knowledge, no data on treatment with mycophenolate mofetil (MMF) are available in genetic NS. Unfortunately, in a randomized US study comparing MMF with cyclosporine, genetic testing was not performed (36). In contrast, data on treatment with calcineurin inhibitors (mainly CsA) in cohorts with genetic SRNS have been published; in most of these studies, genetic diagnosis was confirmed *post hoc*, sometimes after years. Unfortunately, response to treatment interventions is often not detailed and sometimes interpretations are superficial.

In two studies from Buscher et al. (12, 13), the vast majority of patients with genetic FSGS did not achieve remission with cyclosporine. In the first study on 91 patients, mutation in 1 of 6 genes studied were detected in 52% of patients. None of the patients with mutations showed a complete response to CsA, but two patients with Wilms tumor suppressor gene 1 (*WT1*) mutation showed partial remission. The response rate of CSA was significantly better without mutations in podocyte genes (68 vs. 17%, *p* < 0.005). In the second, larger retrospective multicenter study by Buscher et al., 131/231 patients with SRNS had an identified genetic diagnosis, including 60 patients with congenital NS; of these, 63 (48%) patients had received CSA. 2 patients (1 with congenital NS, CNS) entered complete and 5 partial remission (none with CNS). In summary, this series documents a partial or complete response to CSA in 19% of hereditary SRNS (with no details on genotype stated) and 1.7% of congenital NS.

In a recent report from the Podonet consortium, 906/1,354 patients with SRNS were treated with immunosuppressants (380 with one, 173 with two, and 59 with three or more different, respectively) (11). Among 74 patients with documented genetic diagnosis, two patients had complete and 8 partial remission, respectively. Another four patients entered partial remission with a combination of CSA and RAAS inhibitor. Thus, in this series, a total of 14/74 (19%) patients with genetic SRNS seemed to have a response to immunosuppression.

**Figure 1:**
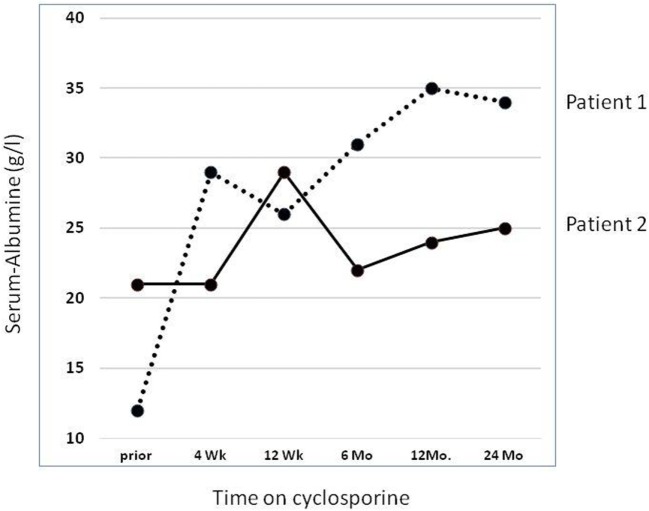
Effect of cyclosporine treatment on evolution of serum albumin (sAlb) levels. Detailed data on two patients reported by Klaassen et al. (37). Dashed line: Patient 1 with compound heterozygous *NPHS1* mutation (c.928G > A, p.Asp310Asn; c.2816-3T > G, p.?) rapidly showed complete remission with concomitant normalization of sAlb. Solid line: Patient 2 with a homozygous *NPHS2* mutation (NPHS2: c.467dupT, p.Leu156Phefs*11) achieved partial remission and albumin infusions were discontinued. He reached ESRD after 5.0 years.

In our own retrospective single center study (37) of nine SRNS patients with an identified genetic diagnosis (excluding patients with CNS, *WT1* mutations, and syndromic NS), we observed a partial (2 patients with *NPHS2* mutations) or complete (one patient with compound heterozygous *NPHS1* and one with a dominant *ACTN4* mutation, respectively) remission. Thus 4/9 (44%) showed some response to CSA; Figure 1 demonstrates evolution of serum-albumin levels in two patients with a complete and partial response, respectively.

Currently, no controlled data are available as to how long CSA treatment in genetic forms of SRNS should be continued, if no response is documented. The series by Klaassen et al. (12, 13, 37), however, show that most patients with complete remission responded after a median of 2 months, so that this treatment period seems to be a minimum. It is currently unknown, whether patients with distinct mutation, e.g., in *WT1* or *NPHS2* show a differential response.

## Experiences in Specific Genetic Disorders

### *WT1*-Mutations

The Wilms Tumor Suppressor Gene 1 (WT1) is a transcription factor with many functions, among them transcriptional as well as tumor-suppressor activities. WT1 plays a pivotal role in early urogenital and kidney development (38), in adults, it continues to be a key regulator of podocyte function (39). Mutations in *WT1* mostly occur as spontaneous heterozygous germline mutations, but familiar cases are also described. Type and location of specific mutations allow for limited prediction of clinical course, histology, and comorbidities (40). By far, not all patients show all symptoms of the classical syndromal descriptions associated with *WT1* mutation: Denys–Drash syndrome described in patients with missense mutations, it includes DMS, SRNS rapidly progressing to end-stage renal disease, XY disorder in sex development with complete gonadal dysgenesis, and a high risk of developing Wilms’ tumor. Frazier Syndrome is typically caused by mutations affecting the canonic donor splice site of intron 9, patients present with streak gonads, and are at high risk of developing gonadoblastoma.

There is evidence from studies with small patient numbers that early initiation of treatment with CsA in combination with RAAS inhibitors can lead to favorable response of NS in patients with *WT1* mutations (41). Gellermann et al. report on three children with WT1 mutations and FSGS in whom long-term reduction of proteinuria could be achieved through treatment with CSA in combination with steroids and RAAS inhibitors while maintaining normal renal function (42). Wasilewska et al. describe a patient with *WT1* mutation and DMS on histology in whom nephrotic range proteinuria resolved after initiation of treatment with CSA and enalapril (43). Buscher et al. report two patients affected by NS due to mutations in *WT1* showing a partial response to CsA with a reduction of proteinuria and normalization of sAlb (13). Unfortunately, in their 2016 study, Buscher et al. do not give details on their patients with *WT1* mutations treated with CSA (12). Further studies are needed to define in more detail which patients with *WT1* mutations (type of mutation and histological changes) can benefit from immunosuppressive treatment with CSA. Mechanisms mediating the benefits of CSA, other than stabilizing podocyte cytoskeleton, in patients with disturbed WT1 expression also need further elucidation (41). Collection of patients with *WT1*-mutations in a prospective registry has been initiated by the German Society of Pediatric Nephrology (GPN).

### Coenzyme Q_10_ (CoQ_10_)-Deficiency

CoQ_10_, also known as ubiquinone, is involved in many essential cellular processes, especially in the mitochondria. Its biosynthesis requires at least 15 genes. So far, mutations in eight of these genes have been found to cause primary CoQ_10_ deficiency, which results in diseases with variable age of onset. Associated clinical phenotypes are ranging from a multisystem disease to nephropathy or isolated central nervous system disease (myopathy or cerebellar ataxia) (44). Nephropathy can result in steroid resistant NS and loss of renal function in an isolated form (45) or in combination with sensorineural deafness (46) or other neurological symptoms (47).

In contrast to most mitochondrial respiratory chain disorders, for which there is no effective treatment, patients with primary CoQ_10_ deficiency partially respond to oral CoQ_10_ supplementation (48). *In vitro* data and first clinical experiences suggest that high-dose oral treatment with CoQ_10_ has the potential to stop the progression of encephalopathy, muscular symptoms, and NS and even induce remission, if initiated early enough. Heeringa et al. (46) describe proteinuria and hearing loss in patients with coenzyme Q_10_ biosynthesis monooxygenase (*COQ6)*-mutations. *In vitro*, apoptosis caused by *COQ6*-knockdown was partially reversed by CoQ_10_ treatment. The authors report a positive response to treatment with CoQ_10_ and RAAS inhibitors in two children with mild disease. Ashraf et al. (49) describe that mutations in the aarF domain containing kinase 4 gene (ADCK4) leads to CoQ_10_ deficiency causing SRNS. Knockdown of *ADCK4* in podocytes resulted in decreased migration, which was reversed by CoQ10 addition. Indeed, one individual with *ADCK4*-mutation was successfully treated with CoQ_10_ supplementation. Once severe kidney or neurological damage is established, this cannot be reversed (47, 48).

### Gene Therapy in Genetic Forms of SRNS

So far, no successful gene therapeutic approaches have been reported for hereditary forms of SRNS. However, a mouse model for NPHS2 has recently been reported (50) and may have important consequences also for the development of specific molecular treatment approaches. Other investigations have indicated a role of specific miRNAs in the etiology of FSGS, which may be amenable to specific treatment (51). Taken together, studies into the molecular biology of SRNS have a yet undiscovered potential to develop new treatment modalities and potentially a cure for certain genetic forms of SRNS.

## Summary

**Figure 2:**
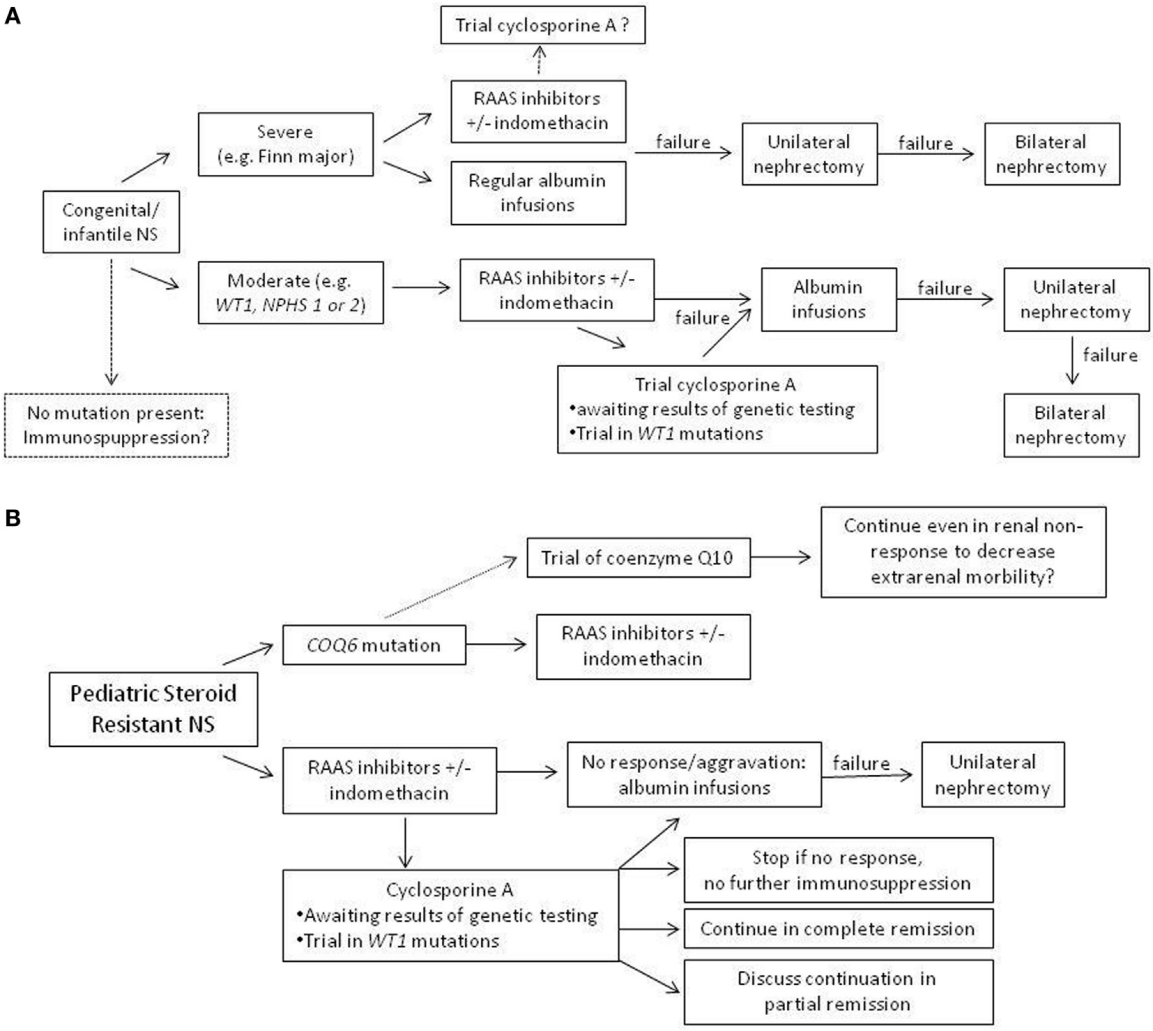
Suggestion of an algorithm for treatment in genetic nephrotic syndrome (NS). **(A)** Congenital and infantile NS with positive testing results. **(B)** Pediatric steroid-resistant nephrotic syndrome, where often, results of genetic testing are not available immediately.

Although no adequate systematic treatment studies have been performed in patients with genetic forms of NS, choice of treatment needs to consider clinical factors (e.g., severity of clinical presentation and age of presentation). In a clinically stable patient with congenital/infantile NS, use of an RAAS inhibitor could be first choice. In neonates and infants, captopril has been most frequently used and can be titrated best. In severe cases, a combination of RAAS inhibitor with indomethacin seems appropriate in order to avoid (unilateral or even bilateral) nephrectomy, which is an effective option in severely affected individuals. In steroid-resistant NS patients, most patients with Mendelian forms seem resistant to immunosuppressive treatment. Yet, several studies have shown that in some patients, a complete or partial remission with CSA can be achieved also in hereditary SRNS, including patients with infantile nephrotic syndrome (Figure 2). Therefore, in our opinion, a therapeutic trial with CSA in genetic SRNS is justified, since especially CSA has been shown to exert a relevant podocyte stabilizing effect, which may reduce proteinuria in some patients. In the future, more clinical studies of optimal treatment of genetic SRNS are necessary, also evaluating the impact of confounding factors, such as genotype/phenotype correlations, impact of attenuation of proteinuria on rate of progression into ESRD, and others. Ultimately, gene therapy will hopefully be available in the future offering a specific cure of (some) genetic causes of steroid resistant NS.

## Author Contributions

MK wrote the first draft of the manuscript; AL wrote sections of the manuscript. Both authors contributed to manuscript revision, read, and approved the submitted version.

## Conflict of Interest Statement

The authors declare that the research was conducted in the absence of any commercial or financial relationships that could be construed as a potential conflict of interest.
